# Phase II study of BKM120 in patients with advanced esophageal squamous cell carcinoma (EPOC1303)

**DOI:** 10.1007/s10388-022-00928-3

**Published:** 2022-07-29

**Authors:** Takashi Kojima, Ken Kato, Hiroki Hara, Shunji Takahashi, Kei Muro, Tomohiro Nishina, Masashi Wakabayashi, Shogo Nomura, Akihiro Sato, Atsushi Ohtsu, Toshihiko Doi

**Affiliations:** 1 Department of Gastrointestinal Oncology, National Cancer Center Hospital East, 6-5-1, Kashiwanoha, Kashiwa, Chiba, 277-8577 Japan; 2 Department of Gastrointestinal Medical Oncology, National Cancer Center Hospital, Chuo-ku, Tokyo, Japan; 3 Saitama Cancer Center Hospital, Saitama, Japan; 4 Cancer Chemotherapy Center, Cancer Institute Hospital, Tokyo, Japan; 5 Aichi Cancer Center Hospital, Nagoya, Japan; 6 Shikoku Cancer Center, Matsuyama, Japan; 7 Clinical Research Support Office, National Cancer Center Hospital East, Kashiwa, Japan

**Keywords:** NVP-BKM120, Esophageal Neoplasms, Phosphatidylinositol 3-Kinases

## Abstract

**Background:**

PI3K/AKT/mTOR pathway is frequently overactive in esophageal squamous cell carcinoma (ESCC), making it an attractive treatment target. BKM120 is an oral pan-class I PI3K inhibitor with promising activity in several cancers. We prospectively investigated efficacy, safety, and biomarkers of BKM120 in advanced ESCC. We conducted a multicenter phase II study of BKM120 monotherapy in patients with pretreated advanced ESCC.

**Methods:**

BKM120 (100 mg/day) was administered orally in a 28-day cycle. The primary end point was disease control rate (DCR). Tumor samples for all patients were collected for gene alteration analysis in a comprehensive genomic profiling assay.

**Results:**

Of 42 patients enrolled, 20 had stable disease and two had confirmed partial response. One ineligible patient was excluded from the primary analysis, which met the primary end point (DCR 51.2%; 95% confidence interval [CI], 35.1–67.1). In the 42 patients, median progression-free survival and overall survival were 2.3 (95% CI 1.8–3.2) and 9.0 (95% CI 6.5–11.4) months, respectively. Common grade 3 or 4 adverse events were rash, anorexia, hyponatremia, and abnormal hepatic function; profiles of these events in this study were similar to those in previous studies of BKM120 monotherapy. No treatment-related deaths occurred. PI3K pathway activation was observed in patients with good clinical response.

**Conclusions:**

BKM120 monotherapy showed promising efficacy and a manageable toxicity profile even in patients with pretreated advanced ESCC. This study showed the potential target PI3K for ESCC, and further confirmatory trial will be necessary to confirm it. Unique ID issued by UMIN: UMIN 000011217.

## Introduction

Esophageal cancer is the eighth most common cancer and the sixth most common cause of cancer-related death worldwide [1].

Patients with recurrent or metastatic esophageal squamous cell carcinoma (ESCC) usually have a particularly poor prognosis, with a median overall survival (OS) from 6 to 10 months [2, 3], and there are few chemotherapeutic agents available. Commonly used agents include 5-fluorouracil, platinum agents, taxanes, and anti-PD-1 antibody. Although molecularly targeted agents substantially improve the outcome of several types of cancers, unfortunately no agent has shown clinically significant benefit in ESCC. While there is an unmet medical need in ESCC, clinical developments for new agents have not progressed because of ethnic differences in history, economic issues, and a lack of driver gene mutation.

EGFR overexpression occurs in 32–86% of ESCC. Although EGFR represents one of the most investigated molecular targets in ESCC, in recent clinical trials, combination treatment with anti EGFR antibody and radiotherapy or conventional chemotherapy failed to show the additional treatment efficacy [4–6]. It had been reported that EGFR overexpression might be the relevant biomarker for activity of anti EGFR antibody, and some report suggested that PI3KCA/PTEN gene deregulation significantly correlated with an impaired response to anti EGFR antibody [7, 8].

The PI3K/AKT/mTOR pathway within cancer cells is important for tumor cell growth, proliferation, survival, motility, and metastasis. Recently, it was reported that the PI3K pathway also plays a role in leukocyte recruitment and activation, vascular integrity maintenance, and other aspects of the tumor microenvironment [9]. An activating *PIK3CA* mutation or amplification has been observed from 2.2 to 21% of patients with ESCC [10–13]. PIK3CA mutation may be possible a potential target molecule in ESCC.

BKM120 is a potent and highly specific oral class I phosphoinositide 3-kinase (PI3K) inhibitor that belongs to the 2,6-dimorpholino pyrimidine derivative family. BKM120 has demonstrated antiproliferative, proapoptotic, and antitumor activity in a variety of cell lines and xenograft models from cancers [14].

BKM120 demonstrated broad antitumor activity in a phase I clinical study [15–17]. As a potential predictive biomarker for PI3K inhibitors, phosphatidylinositol 4,5-bisphosphate 3-kinase catalytic subunit alpha isoform (*PIK3CA*) mutations have been suggested in a review of early-phase clinical trials in various solid cancers [18].

In a phase I study involving an mTOR inhibitor (RAD001), one patient with ESCC demonstrated a partial response [19]; similarly, head and neck cancer patients demonstrated a partial response with BKM120 [15]. The results of a randomized phase II study in patients with platinum-treated head and neck cancer reported that BKM120 plus paclitaxel improves progression-free survival (PFS) compared with paclitaxel alone [20]. Therefore, we conducted this multicenter phase II study of BKM120 in patients with ESCC.

The aim of this phase II study was to assess the efficacy and safety of BKM120 monotherapy in patients with ESCC. We therefore performed exploratory biomarker analyses by next-generation sequencing-based comprehensive genomic profiling (FoundationOne) to predict the efficacy of BKM120 in this patient population.

## Patients and methods

### Patient population

Patients had to be aged ≥ 20 years and have the following characteristics: (1) histologically confirmed unresectable or recurrent ESCC refractory to one or two standard regimens that contained fluoropyrimidine and platinum derivatives; (2) discontinued the last prior chemotherapy before enrollment because of radiologic disease progression or an adverse event; (3) evaluable disease as defined by Response Evaluation Criteria in Solid Tumors (RECIST, version 1.1); (4) Eastern Cooperative Oncology Group (ECOG) performance status of ≤ 2; (5) adequate bone marrow, hepatic, and renal function; and (6) fasting plasma glucose levels of ≤ 120 mg/dL. A tumor specimen (primary or metastatic) was acquired from archival material or fresh biopsy.

The key exclusion criteria were treatment with CYP3A4 modifier drug ≤ 1 week before starting BKM120, clinical manifestation of diabetes mellitus, clinically documented depression or anxiety on the Patient Health Questionnaire-9 and Generalized Anxiety Disorder Screener-7 mood scales, and previous treatment with PI3K/AKT/mTOR inhibitors.

### Study design

This multicenter, phase II, open-label, single-arm study was conducted to evaluate the efficacy and safety of BKM120 monotherapy in ESCC at six Japanese institutions. The ethics committees (institutional review board) of the participating institutions and regulatory authorities approved this study. All patients provided informed consent. The study followed the Declaration of Helsinki and Good Clinical Practice guidelines. The study was registered at the UMIN clinical trials registry (http://www.umin.ac.jp/ctr/index-j.htm; registration number UMIN000011217).

### Study treatment and assessment

All patients received BKM120 (100 mg/day) until disease progression, unacceptable toxicity, or withdrawal of consent. In the case of adverse events (AEs) or toxicity considered to be related to BKM120, dosing was delayed or reduced according to an algorithm outlined in the study protocol.

Each investigator evaluated antitumor response at 4 and 8 weeks after treatment initiation and then every 4–6 weeks in accordance with RECIST guidelines. When treatment was discontinued for any reason other than progressive disease, follow-up imaging was performed according to the planned schedule until disease progression or subsequent anticancer treatment.

The primary end point was the investigator-assessed disease control rate (DCR), which was defined as the proportion of patients with the best overall response of complete response (CR), partial response (PR), or stable disease (SD) based on the RECIST guidelines.

DCR was analyzed in the per-protocol set over 8 weeks (PPS8W), which included a subset of eligible patients who fulfilled the minimum exposure requirement (relative dose of ≥ 0.5) until 8 weeks or who experienced progression before the minimum exposure requirement without any major protocol deviation. Imaging data of patients with investigator-assessed CR, PR, or SD (best response) were independently reviewed by a single radiologist who had 5 years or more of subspecialty experience in diagnostic oncologic radiology.

The secondary efficacy end points were objective response rate (ORR), OS, and PFS, all analyzed in the per-protocol set, which included a subset of eligible patients. ORR was defined as the proportion of patients with the best overall response of CR or PR based on the RECIST guidelines. OS was defined as the time from enrollment until death from any cause. PFS was defined as the time from enrollment until tumor progression, as determined by investigator assessment, or death from any cause.

Safety analysis was performed in the safety population (SP), which comprised patients who received BKM120 monotherapy. AEs were assessed according to the Common Terminology Criteria for AEs (version 4.0).

### Statistical plan

The study used Simon’s minimax two-stage design with a one-sided α level of 10% and power of 90%. A DCR of 40% was considered nonpromising, whereas a DCR of 60% was considered promising in this population. In the first stage, 28 PPS8W patients were to be enrolled, and termination of the trial was considered if ≤ 11 patients achieved CR, PR, or SD. In the second stage, 13 additional PPS8W patients were to be enrolled. Of the total 41 PPS8W patients, the null hypothesis of DCR of 40% would be rejected if ≥ 21 patients achieved CR, PR, or SD.

The ORR and DCR, and their exact binomial 95% confidence intervals (CI), were estimated. The Kaplan–Meier method was used to analyze PFS and OS, with estimates for median time-to-event end points and their 95% CIs. All statistical analyses were performed using SAS software (release 9.4; SAS Institute, Inc., Cary, NC).

### Biomarker analysis

Exploratory biomarker analysis was performed on a subset of tumor samples, and sufficient material was available. (In cases where tumor samples could not be collected by biopsy, but archival tumor samples were available, the use of archival tumor samples was also permitted.) Next-generation sequencing-based comprehensive genomic profiling was performed on all formalin-fixed paraffin-embedded tissues using a hybrid capture-based next-generation sequencing platform (FoundationOne; Foundation Medicine, Cambridge, MA) at a Clinical Laboratory Improvement Amendments–certified, New York State and College of American Pathologists–accredited laboratory (Foundation Medicine) on the Illumina HiSeq2500 instruments (Illumina, Inc., San Diego, CA).

## Results

### Patient characteristics

From August 2013 to August 2016, 42 patients (median age 62.5 years; ECOG performance status 0/1 = 28/14) were enrolled in this study. After enrollment, one patient treated with BKM120 was found to be ineligible because of three prior treatment regimens received, and the patient was excluded from PPS8W. In the first stage, DCR was 53.6% (15 of 28 PPS8W patients achieved SD). Therefore, we enrolled an additional 13 patients in the second stage. All 42 enrolled patients were assessable as the SP.

All patients had received fluoropyrimidine and platinum agents, whereas 31 patients (73.8%) had received taxanes. A total of 22 patients had previously undergone esophagectomy (52.4%; Table 1).

**Table 1:** Patient Characteristics (n = 42)

Characteristic	*n*	(%)
Sex, male	38	(81.0)
Age, median (years)	62.5	
ECOG performance status		
0	28	(66.7)
1	14	(33.3)
Previous esophagostomy		
Yes	22	(52.4)
No	20	(47.6)
Previous radiotherapy		
Yes	24	(57.1)
No	18	(42.9)
No. of prior regimens		
1	12	(28.6)
2	29	(69.0)
3	1	(2.4)
Prior treatment		
Fluoropyrimidine + platinum	35	(83.3)
Taxanes	24	(57.1)
DCF^a^	10	(23.8)
Immune checkpoint inhibitor	3	(7.1)

Abbreviation: *ECOG* Eastern Cooperative Oncology Group

^a^DCF: 5-fluorouracil + cisplatin + docetaxel

### Exposure to chemotherapy

Patients underwent treatment for a median duration of 57 days (range 5–225 days); 17 patients (40.5%) required a dose reduction and 27 (64.3%) interrupted their treatment. The relative dose intensity of BKM120 was maintained in all treatment periods (median 95.7%; range 42.7–100%), and most patients (78.6%) were treated until disease progression (Table 2).

**Table 2:** Exposures (n = 42)

Relative Dose Intensity	
At 8 weeks, median, % (range)	96.4 (48.6–100.0)
All periods, median, % (range)	95.7 (42.7–100.0)
No. of days, median (range)	57 (5–225)
Dose reduction, *n* (%)	17 (40.5)
Interruption, *n* (%)	27 (64.3)
Reasons for discontinuation	
Disease progression, *n* (%)	33 (78.6)
AEs, *n* (%)	4* (9.5)
Patient refusal, *n* (%)	3 (7.1)
Other, *n* (%)	2 (4.8)

Abbreviation: *AEs* adverse events

^*^Maculopapular rash (1), syncope (1), fatigue and GGT increased (1), hepatic disorder (1)

### Antitumor activity

**Fig. 1:**
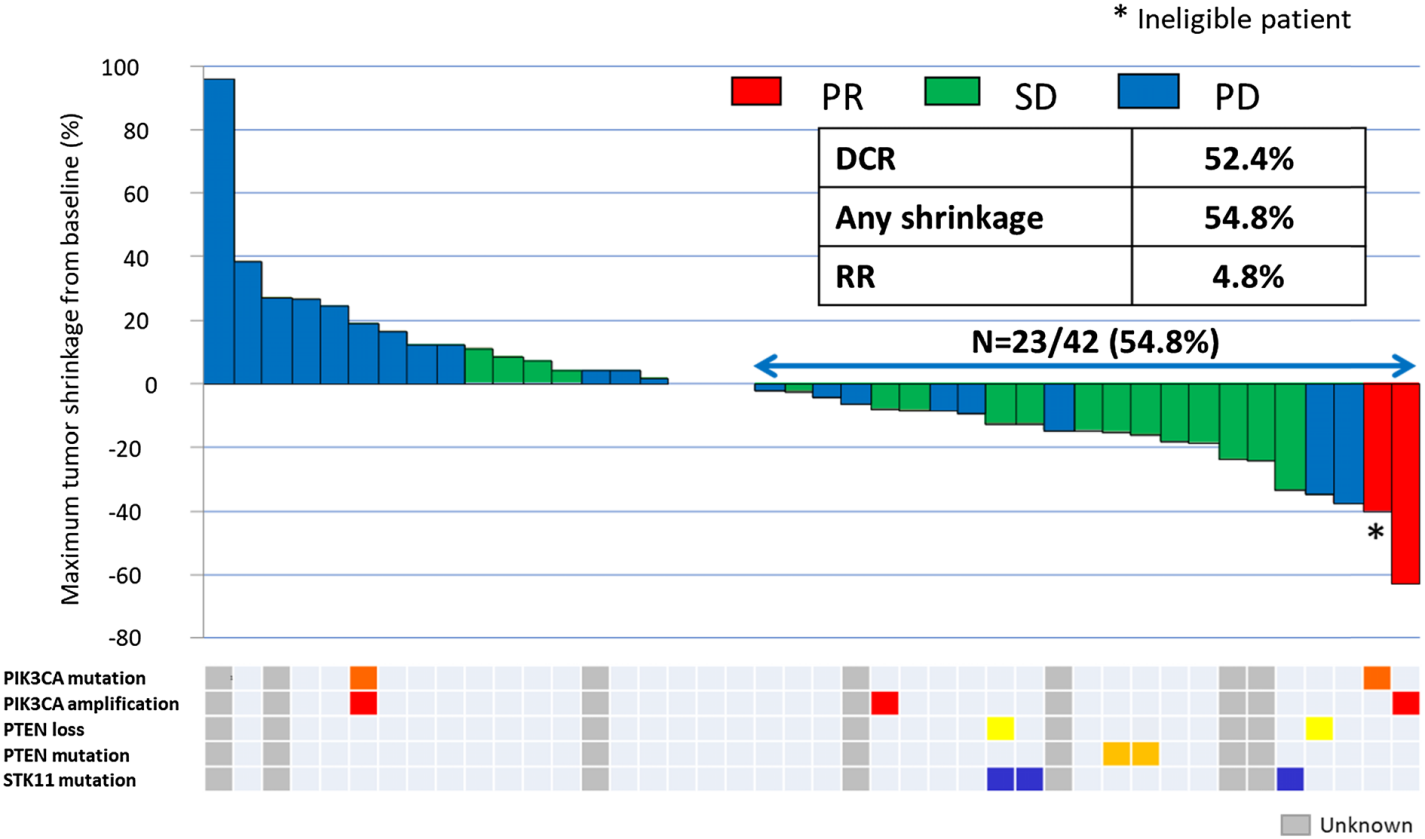
Waterfall plot (*n* = 42). Radiologic response to BKM120 with corresponding status of tumor PIK3CA, PTEN, and STK11. *DCR* disease control rate, *PD* progressive disease, *PIK3CA* phosphatidylinositol 4,5-bisphosphate 3-kinase catalytic subunit alpha isoform, *PR* partial response, *PTEN* phosphatase and tensin homolog, *RR* relative risk, *SD* stable disease, *STK11* serine/threonine kinase 11

The best overall responses were evaluated by investigators and an independent radiologic review (Table 3). A total of 42 patients, including one ineligible patient excluded from the primary analysis, were evaluable for response by investigator review of target lesion radiologic assessments. Of the 42 patients, two achieved confirmed PR, the ORR was 4.8%, and DCR was achieved by 52.4% of all patients. The investigators’ assessments of the PPS8W population revealed that one patient achieved PR and 20 achieved SD, which corresponded to an investigator-assessed DCR of 51.2% (95% CI 35.1–67.1). A total of 21 patients achieved DCR, which exceeded the threshold of 20 patients. Although no patient achieved CR, the tumor size decreased in 54.8% of patients (Fig. 1).

**Table 3:** Overall response

Best response	By the investigators				Central review			
	*n* = 41 PPS8W		*n* = 42		*n* = 41 PPS8W		*n* = 42	
	*n*	%	*n*	%	*n*	%	*n*	%
CR	0	0	0	0	0	0	0	0
PR	1	2.4	2	4.8	3	7.3	4	9.5
SD	20	48.8	20	47.6	18	43.9	18	42.8
PD	20	48.8	20	47.6	20	48.8	20	47.6
DCR (95% CI)	51.2 (35.1–67.1)		52.4 (36.4–68.0)					
RR (95% CI)	2.4 (0.1–12.9)		4.8 (0.6–16.2)					

*CI* confidence interval, *CR* complete response, *DCR* disease control rate, *PD* progressive disease, *PPS8W* per-protocol set over 8 weeks, *PR* partial response, *RR* relative risk, *SD* stable disease

During the independent central review for 33 patients, the radiologist concluded that two patients who were classified as having SD by the investigator assessment actually had PR. Therefore, the relative risk according to the independent central review was 9.5%.

**Fig. 2:**
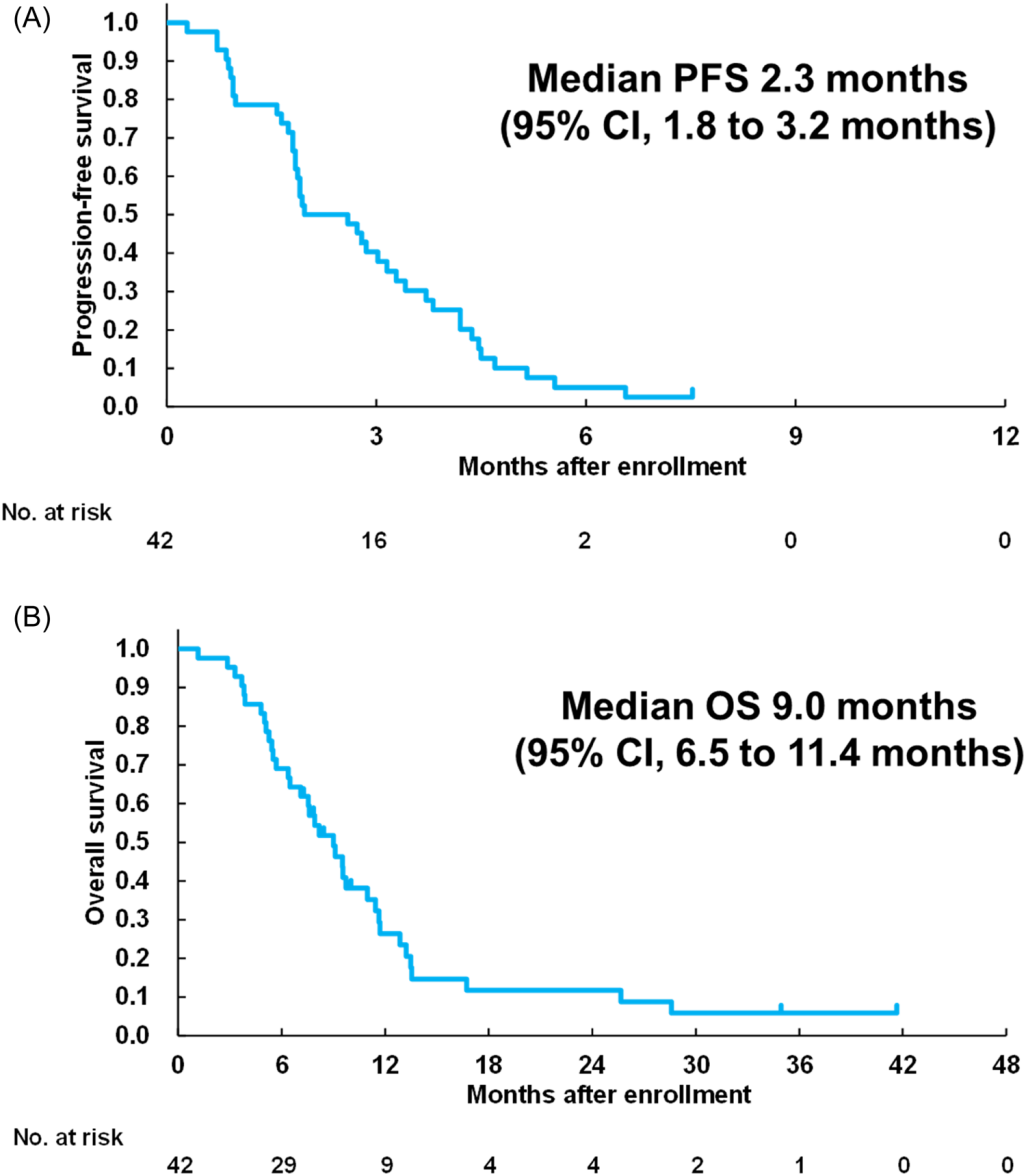
**A** Progression-free survival. **B** Overall survival. *n* = 42. *CI* confidence interval, *OS* overall survival, *PFS* progression-free survival

At the data cutoff date (March 6, 2017), all patients except one had experienced disease progression, and one had PR. The median investigator-assessed PFS was 2.3 months (95% CI 1.8–3.2 months; Fig. 2A). After a median follow-up of 9.2 months, the median OS was 9.0 months (95% CI 6.5–11.4 months; Fig. 2B).

### Safety findings

BKM120 monotherapy was well tolerated by all patients. The most common grade 3 or 4 AEs were maculopapular rash (9.5%), anorexia (7.1%), aspartate aminotransferase increase (4.8%), alanine aminotransferase increase (4.8%), diarrhea (4.8%), fatigue (4.8%), hyperglycemia (2.4%), and oral mucositis (2.4%), the profiles of which were similar to those of previous studies of BKM120 monotherapy (Table 4). No treatment-related deaths occurred.

**Table 4:** Major adverse events (> 10% of any grade; *n* = 42)

Adverse event	Any grade, *n* (%)	Grade 3/4, *n* (%)
Hyperglycemia	24 (57.1)	1 (2.4)
Maculopapular rash	20 (47.6)	4 (9.5)
Anorexia	13 (31.0)	3 (7.1)
Aspartate aminotransferase increase	13 (31.0)	2 (4.8)
Oral mucositis	13 (31.0)	1 (2.4)
Alanine aminotransferase increase	11 (26.2)	2 (4.8)
Nausea	10 (23.8)	0
Diarrhea	8 (19.0)	2 (4.8)
C-peptide increase	8 (19.0)	0
Malaise	8 (19.0)	0
Anxiety	7 (16.7)	0
Dry skin	7 (16.7)	0
Fatigue	6 (14.3)	2 (4.8)
Vomiting	6 (14.3)	0
Depression	5 (11.9)	0
Platelet count decrease	5 (11.9)	0
Dysgeusia	5 (11.9)	0
Lipase increase	4 (9.5)	3 (7.1)
Alkaline phosphatase increase	3 (7.1)	0
Creatinine increase	3 (7.1)	0
Fever	3 (7.1)	0
Hypertension	3 (7.1)	0
Hyponatremia	3 (7.1)	3 (7.1)
Photosensitivity	3 (7.1)	1 (2.4)
Palmar-plantar erythrodysesthesia syndrome	3 (7.1)	0

No treatment-related deaths were observed

### Biomarker analysis

Thirty-five tumor samples were found to be evaluable; eight (23%) had PI3K pathway alterations (Fig. 1), including *PIK3CA* mutation (*n* = 2), *PIK3CA* amplification (*n* = 3), phosphatase and tensin homolog (*PTEN*) mutation (*n* = 2), and *PTEN* loss (*n* = 2). The missense mutation p.F354L in exon 8 of the serine/threonine kinase 11 (*STK11*) gene was identified in three cases.

Waterfall plots are shown in Fig. 1. The first patient with PR had *PIK3CA* amplification and experienced a 63% reduction in target lesion size. Time to response was 97 days and duration of response was 132 days. The second patient with PR had *PIK3CA* mutations and experienced a 40% reduction in target lesion size. Time to response was 29 days and duration of response was 114 days.

## Discussion

This study was designed to evaluate the efficacy and safety of BKM120 monotherapy in patients with recurrent or metastatic ESCC. The primary end point of the study (i.e., a promising DCR) was met by 51.2% (95% CI 35.1–67.1) of pretreated patients with ESCC. In addition, 54.8% of patients demonstrated tumor shrinkage from baseline. The median PFS was 2.3 months, and the median OS was 9.0 months.

The choice of DCR as the primary end point in this study was considered appropriate because it reflects clinical practice, where progression usually necessitates a change of treatment; also, its use is appropriate in a proof-of-concept study in the second- and third-line settings. Patients in this study were previously treated; nearly 70% received BKM120 as a third-line therapy.

BKM120 was generally well tolerated, and no new safety concerns were identified in the study. The three main categories of AEs suspected to be related to BKM120 were hyperglycemia, liver function abnormalities, and mood disorders. The most common grade 3 or 4 AEs were anorexia, rash, hyponatremia, lipase increase, and abnormal hepatic function (including increased transaminase levels), the profiles of which were similar to those of previous studies of BKM120 monotherapy [15, 21].

Preliminary signs of clinical efficacy were observed in this study, with two patients (4.8%) exhibiting PR. The DCR reported here (52.4%) was similar to rates observed with BKM120 in other patient populations: 41% and 40% in Western and Japanese patient populations, respectively [15, 17].

Among patients with a known gene alteration status, PI3K activation (defined as *PIK3CA* mutation, *PIK3CA* amplification, *PTEN* mutation, and *PTEN* loss) was exhibited in 19% (8/42) of patients with ESCC. The observed frequency of PI3K pathway alterations in ESCC was similar to that reported by a previous study of The Cancer Genome Atlas, in which approximately 21% of 90 patients exhibited *PIK3CA* mutations or *PTEN* alterations [22]. In our study, seven of eight patients with PI3K activation demonstrated tumor shrinkage from baseline, and two patients achieved PR.

Although it is possible that BKM120 will show greater efficacy in patients with activated PI3K signaling, this study contained too few patients to determine any correlation between mutation status and response. The correlation between PI3K activation status and clinical response is not conclusive based on results from previous trials of BKM120 and other PI3K inhibitors [16, 17, 23–26]. Further research is needed to determine if PI3K activation status is a predictor of BKM120 response and whether selecting for patients with PI3K activation could improve outcomes with BKM120.

In our study, all three patients with mutations in *STK11* demonstrated tumor shrinkage and had a missense mutation at amino acid 354, leading to conversion of the wild-type residue phenylalanine to a leucine (*STK11*, p.F354L, c.1062C > G). The serine/threonine kinase STK11 (also called LKB1) activates AMP-activated protein kinase (AMPK) and negatively regulates the mTOR pathway in response to changes in cellular energy levels [27]. STK11 acts as a tumor suppressor in cancer because loss of function promotes proliferation and tumorigenesis [28]. The F354L mutation has been shown to impair STK11-mediated AMPK activation and lead to increased mTOR signaling [29].

As with many early-phase studies with other PI3K inhibitors in advanced solid tumors, no association was identified between the extent of tumor shrinkage or best overall response, as per investigator assessment, and the tumor molecular alterations analyzed. This lack of association could be due to several factors, such as the small sample size, time lag between the archival sample used for pathway analysis and the time of patient entry in the trial, and heterogeneous patient population.

In conclusion, BKM120 monotherapy showed promising efficacy and a mild toxicity profile in patients with pretreated advanced ESCC. PI3K inhibitors, such as BKM120, are worthy of further evaluation in confirmatory studies.
